# Resilient Parents… Resilient Communities: A Pilot Study Trialing the Bounce Back and Thrive! Resilience-Training Program With Military Families

**DOI:** 10.3389/fpsyg.2021.651522

**Published:** 2021-07-19

**Authors:** Cynthia Mikolas, Ashley Pike, Chelsea Jones, Lorraine Smith-MacDonald, Melina Lee, Hope Winfield, Jennifer Griffiths, Ryan Perry, David M. Olson, Alexandra Heber, Joanne Olson, Phillip R. Sevigny, Suzette Brémault-Philips

**Affiliations:** ^1^Military Family Resource Centre, Canadian Forces Base Edmonton, Edmonton, AB, Canada; ^2^Heroes in Mind, Advocacy and Research Consortium (HiMARC), Faculty of Rehabilitation Medicine, University of Alberta, Edmonton, AB, Canada; ^3^Department of Occupational Therapy, Faculty of Rehabilitation Medicine, University of Alberta, Edmonton, AB, Canada; ^4^1 Field Ambulance, Canadian Forces Health Services, Canadian Forces Base Edmonton, Edmonton, AB, Canada; ^5^Faculty of Rehabilitation Medicine, University of Alberta, Edmonton, AB, Canada; ^6^Faculty of Nursing, University of Alberta, Edmonton, AB, Canada; ^7^Royal Canadian Chaplain Service, Canadian Forces Health Services, Canadian Forces Base Edmonton, Edmonton, AB, Canada; ^8^Department of Obstetrics & Gynecology, Faculty of Medicine and Dentistry, University of Alberta, Edmonton, AB, Canada; ^9^Department of Psychiatry, University of Ottawa, Ottawa, ON, Canada; ^10^Department of Educational Psychology, Faculty of Education, University of Alberta, Edmonton, AB, Canada

**Keywords:** military families, resilience, training, mental health, child development, skill building, well-being, program evaluation

## Abstract

**Introduction:**

The resilience of Canadian military families (CMFs) – the main support of the Canadian Armed Forces service members (SMs) – is imperative. The Canadian Armed Forces aims to ensure that SMs and their families are resilient and SMs ready to respond when called upon for combat, peacekeeping or pandemic/disaster-response. Family concerns, however, can realistically distract SMs from the mission, potentially compromising themselves, their unit and the mission. Resilience-training programs such as Bounce Back and Thrive! (BBT) can help families manage the realities of military life.

**Objective:**

This pilot study aimed to evaluate suitability of BBT implementation by Military Family Resource Centers (MFRCs), including whether BBT: (1) fosters resilience-building among parents, (2) facilitates CMF resilience-building, (3) can be contextualized for CMFs, and (4) supports MFRCs in cultivating a culture of resilience.

**Methods:**

An exploratory qualitative design was used. BBT was offered to parents face-to-face. Participants completed focus groups after the first 6 sessions, final 4 sessions, and one-year post-intervention. Data was thematically analyzed.

**Results:**

Nine military parents participated. Four major themes resulted: (1) military parent resilience-building, (2) CMF resilience-building, (3) BBT program feedback and contextualization, and (4) MFRCs as community resilience hubs.

**Discussion:**

BBT enabled parents to gain a new perspective on resilience, engage in dialogue and intentionally role model resilience skills. Military-specific BBT contextualization and online-delivery formats would increase suitability and access for CMFs. Access to resilience programs delivered through MFRCs would support CMFs. Further research is warranted.

## Introduction

With Canadian military families (CMFs) being the main support of Canadian Armed Forces (CAF) serving members (SMs) (Government of Canada and National Defence, 2017a, 6), enhancing their resilience is imperative. The CAF is committed to ensuring that SMs are strong, resilient and ready to respond when called upon by the national and international community for combat, peacekeeping or disaster-response. Regrettably, concerns of a domestic nature can interfere with SMs’ ability to focus, potentially compromising themselves, their unit and the mission. Resilience-training may enhance CMF resilience and ability to manage military-specific circumstances including mobility, absence/separation, and risk of injury or death) (Cramm et al., 2018; Manser, 2020c), mental health, financial and relational challenges, and realities of relocation [e.g., housing, employment, child-care (Battams and Mann, 2018), academic and social challenges (Manser, 2018b)].

### Demographics of CMFs

In August 2017, CAF reported 66,471 Regular Force (RegF) (85% male; 15% female), 47,135 Reserve Force (ResF) SMs (Manser, 2018c) and 99,716 RegF and 38,398 ResF family members (including spouses, children, and dependents), totaling 251,721 CMF members. Nearly half (47%; 29,601) of RegF SMs posted in Canada have children, with 47% having at least one 0–5-year-old (total = 19,162); 45% at least one 6–12-year-old (total = 19,402), and 37% at least one child aged 13–25 years (total = 11,028) (Manser, 2018a). Each CMF has unique needs (e.g., special needs children, dependent elders), structures (e.g., single member, new families, single parents, dual service couples) and experiences (Manser, 2020a). While some CMFs reside on CAF bases (Manser, 2018a, 26), 85% live in civilian neighbourhoods (Cramm et al., 2015).

### Resilience and CMFs

Resilience enables positive individual, family and community adaptation in the face of adversity (Ungar, 2013; Fisher et al., 2019). While most CMFs are well-supported and resilient, approximately 10% reportedly struggle (Manser, 2018c, 2020b). Constraints related to military life CMF may impact coping abilities (Meadows et al., 2016; Cramm et al., 2018) and contribute to the development of cumulative or prolonged stressors which heighten the risk of family disruption and poor outcomes (Walsh, 2016; Manser, 2018c). Facilitative environments, including formal and informal supports, are central to developing and maintaining resilience (Ungar, 2013). Several factors impact the development of facilitative environments, and in turn the response to stress, including the physical and psychological health of each family member (Saltzman et al., 2016; Walsh, 2016). Children, whose adjustment is linked to parental mental health, can be particularly affected (Conger et al., 2002). Conversely, strong family belief systems, organizational patterns, support systems, and communication and problem sharing can prevent disruption and strengthen family resilience (Meadows et al., 2016; Walsh, 2016) and have long-standing and intergenerational impacts. Stress tolerance or management may vary between individual family members, allowing for the strengths and vulnerabilities of each member to impact the family (Cramm et al., 2018). This is highly impactful in families who experience regular separations and reunifications.

### CAF’s Commitment to Family Resilience

CAF’s *Strong, Secure and Engaged (SSE)* Defence Policy promotes “well-supported, diverse, resilient people and families” (Government of Canada and National Defence, 2017a, 107), and its *Total Health and Wellness Strategy* (*TH&WS*) emphasizes a “Triad of Responsibility” between SMs/CMFs, the Chain of Command and the care provider/team (Government of Canada and National Defence, 2017b, 11). Of particular note are initiatives which focus on strengthening partnerships with Military Family Resource Centers (MFRCs), the CAF’s Royal Canadian Chaplain Service (RCChS), Health Services Group, Personnel Support Programs (PSP) and Health Promotions Service. These specific groups within the CAF were targeted because of their focus on access to psychological and psychosocial services including a range of crisis and peer supports, psychoeducation on health and wellness, and counselling services (Cramm et al., 2015).

Central to the adoption of these SSE initiatives has been the growth and expansion of MFRCs across the country. Currently MFRCs offer varied programs and services focused on family wellness, with a particular focus on offering programs for children and youth that predominantly address mental health concerns and trauma rather than resilience (Manser, 2015; Manser et al., 2016; Chartier, 2019). To date only one resilience program has been offered to CMFs - a condensed version of CAF’s Road to Mental Readiness Program (R2MR). However, the R2MR program is less focused on resilience-building than on mental health awareness and help-seeking.

### CMF Resilience-Training Service Gap

With CAF’s priority being total health, wellness, resilience and operational readiness of SMs and CMFs, the current state of resilience-training for CMFs is sub-optimal. Further efforts are needed to identify resilience-training programs to address this service gap (Cramm et al., 2015; Chartier, 2019). There is also a paucity of resilience-training programs for CMFs with children aged 0-8 years. Given the high quality of evidence illustrating that resilience-training programs enhance individual and collective resilience (e.g., emotional, cognitive, spiritual, physical, familial, social), the development of critical abilities associated with resilience (e.g., emotional regulation, empathy, self-efficacy, realistic optimism and reaching out), and seem to result in healthier relationships, more fulsome lives and reduced susceptibility to depression (Seligman et al., 1995; Reivich and Shatte, 2002; Kordich-Hall and Pearson, 2004), it seems surprising that greater care and attention has not be given to providing resilience-training for CMFs who experience high-levels of family stress.

### Proposed CMF Resilience-Training Program

Bounce Back & Thrive! (BBT) – a group-based psychoeducational resilience-training program for parents of children aged 0–8 years – holds promise for potential implementation across MFRCs. Well-defined, standardized and evidence-informed, BBT aims to help parents build resilience skills and increase their ability to use modeling and child-friendly activities to teach resilience skills to young children through developing or enhancing participants understanding of family belief systems, organizational patterns, and problem solving (Pearson and Kordich-Hall, 2017). As adult coping and thinking styles have been emulated by children as young as age 2 (Pearson and Kordich-Hall, 2017), strengthening parent’s resilience skills during early parenthood supports individual and family resilience (Kordich-Hall, 2014) and is anticipated to have lifelong and intergenerational impacts. BBT is typically offered in a 10-session format (weekly 2-h sessions over 10 weeks). (See Table 1 for an outline of BBT sessions, objectives, and formats).

**TABLE 1:** BBT sessions, objectives, and formats.

	**Session Topics**	**Session Number**		
		**Standard 10-session Format**	**Trialed 6-session Format**	**Trialed 3-session Format**
Adult Skills Focus: Developing caring relationships and role modeling resilience	Resilience and family strengths	1	1	1
	Role modeling resilience, relaxation and noticing thoughts	2		
	Automatic thoughts, reactions and stress	3	2	
	Thoughts and feelings	4		2
	Thinking habits and managing stressful situations	5	3	
	Healthy and unhealthy beliefs	6	4	
Child Application Skills Focus: Facilitating resilience in children	Empathy	7	5	3
	Autonomy, choices and decision-making	8		
	Empowerment	9	6	
	Flexible thinking, hope and optimism	10		

Several BBT program features may make it suitable for consideration across MFRCs. A trauma-informed, Canadian-made program, BBT draws on the evidence-based, gold-standard Penn Resiliency Program (Seligman et al., 1995), and uses experiential learning to foster resilience across multiple domains. BBT also uses similar language to the R2MR program which has been strongly integrated across all elements of the CAF and would align mental health and resilience messaging to both service members and families. This alignment is critical in facilitating acceptance and endorsement of the program from senior leadership of the CAF. Available in several languages including French, it is being trialed online, and has been successfully delivered to diverse Canadian and international trauma-affected populations (e.g., low income, refugee, Indigenous, disaster-exposed), integrated into family services in several Canadian cities and provinces, and introduced at a Labrador MFRC. Evaluations conducted since 2002 have been favourable (Kordich-Hall, 2014; Liberty et al., 2019) and BBT has successfully promoted ‘cultures of resilience’ among organizations and communities (Pearson and Kordich-Hall, 2017, 49).

### Objectives

This pilot study aims to evaluate BBT’s suitability for potential implementation by MFRCs, including whether it: (1) fosters resilience-building and well-being among military parents, (2) facilitates CMF resilience-building, (3) can be contextualized for CMFs, and (4) supports MFRCs in cultivating a culture of resilience.

## Methods

### Study Design

An exploratory qualitative design was employed. Research Ethics Board approval and CAF Surgeon General Endorsement were received prior to study initiation.

### Recruitment and Sampling

Study participants included CMFs associated with a local MFRC and in which at least one parent was a SM or Veteran, and one child was between the ages 0 to 8 years or was expected. Recruitment was conducted in September and October 2019 through the MFRC (i.e., social media, email notifications and posters). At time of recruitment, the local military base was training and deploying, resulting in fewer Service Members being available to participate. Potential BBT participants were directed to a secure REDCap (Harris et al., 2009) site to access study information. Formal consent, health and demographic data, and questionnaire responses were also collected using REDCap.

### Intervention

BBT was offered face-to-face to parents/primary caregivers (*N* = 9) at the MFRC by a RIRO-trained MFRC Social Worker. While BBT is normally offered over 10-sessions, a 6-session (3-h sessions twice a week for 3 weeks), and 3-session format (6-h sessions weekly for 3 weeks) were trialed to accommodate CMF schedules (see Table 1). Adult skills were taught in November 2019 and child application in January 2020.

### Data Analysis

Qualitative data was collected through audio-recorded semi-structured focus groups (FGs; *N* = 6) with participants (*N* = 9) and MFRC staff (*N* = 3) in November 2019, and January and November 2020. Once transcribed, qualitative FG data was thematically analyzed (deductively and inductively) by four research team members. Coding and analysis was facilitated using NVivo 12 software. Deductive analysis was informed by study objectives and program evaluation criteria proposed by CFMWS. Braun and Clarke’s (2006) framework for qualitative thematic analysis guided inductive analysis. Initial coding was conducted independently by team members to ensure inter-rater reliability. Regular team meetings enabled discussion and verification of codes, resolution of discrepancies and determination of final themes and supporting quotes (Lincoln and Guba, 1985).

## Results

### Description of Participants

Nine individuals participated in BBT and completed data collection. The participant sample has a group mean age of 35.11∓ 3.89 years, of which 55.56% were civilians, 55.56 % were married, and 55.56% had college or university degrees. Participants who identified as SMs or Veterans had an average length of service of 12.60 ∓ 5.86 years. 77.78% of participants attended the BBT sessions without their partner. See Table 2 for participant demographics.

**TABLE 2:** Participant Demographics.

**Demographic characteristics**	**Number of participants**
Total Participants	9
**Sex**	
Female	7 (78%)
Male	2 (22%)
**Status**	
Civilian	5 (56%)
Service Member	2 (22%)
Veteran	2 (22%)
**Marital status**	
Married	5 (56%)
Common law	4 (44%)
**BBT attendance**	
Co-parents	2 (22%)
Individual	7 (78%)
**Total children per participant**	
1 child	3 (33%)
2 children	2 (22%)
3 children	3 (33%)
9 children	1 (11%)
**Children aged 0–8 yrs per participant**	
1 child	7 (78%)
2 children	1 (11%)
3 children	1 (11%)
**Highest level of education**	
High school graduate	1 (11%)
Some college/university	3 (33%)
College/university	5 (56%)
SM/Veteran Participants	4
**Rank**	
Officer	1 (25%)
Senior Non-Commissioned Officer	1 (25%)
Junior Non-Commissioned Officer	2 (50%)
**Service Environment**	
Sea	1 (25%)
Land	2 (50%)
Air	1 (25%)
**Enrollment Era**	
2001–2015	3 (75%)
2016+	1 (25%)
**Deployment History**	
Yes	2 (50%)
No	2 (50%)

### Thematic Analysis

Thematic analysis focused on four major themes: (1) military parent resilience-building, (2) CMF resilience-building, (3) BBT program feedback and contextualization, and (4) MFRCs as community resilience hubs. Triangulation of quantitative and qualitative findings indicated that the BBS results had some similarities with themes and quotes A discussion and table of these themes (see Table 3: Major themes and subthemes) follow, including BBS questions supporting qualitative themes.

**TABLE 3:** Major themes and subthemes.

**Theme**	**Subtheme**	**Supporting quote (FG number)**
Military parent resilience- building	Resilience is a learned skill	*“[BBT] made you [see resilience] step by step and pay attention. [A] very clear explanation of resiliency.” (FG1)*
	Resilience requires reflection and awareness	*“What stood out to me was setting aside the time to work on some stuff that I needed to do as a parent. (…) I never took the time to check in or (…) acknowledge what I do and don’t do.” (FG6)*
	Resilience-building requires practice	*“[I]t wasn’t one specific teachable moment that was (…) enlightening [about BBT. Rather resilience] is something you (…) continually need to work at; (…) it’s the time, effort and energy that needs to go into maintaining that skill.” (FG6)*
CMF resilience- building	BBT supports development of collective family resilience	*“[F]ocusing on (…) and being aware of what you and your child’s strengths are and having that much more visible and (…) concrete was really helpful.” (FG2)* *“[W]e talked about connection with our kid. (…) [B]eing able to communicate better (…) would have helped. A year later, it’s much better (…) [H]ad [the BBT] course been there that conversation would have been open maybe a little earlier.” (FG5*)
	Resilience requires reflection on parenting practices	*“[I’m trying to get] rid of assumptions that I’ve made about my kids and how they’ll behave and (…) react to situations. It was giving them a chance to work through things, giving myself a chance to reflect on the skills I have, (…) and always look for the positives and how we can work at things together as a family.” (FG6)*
	Requirement of collective understanding and practice of resilience skills.	*“If we react better on a constant basis, then when the big things happen, we’re better equipped.” (FG1)* *“It gave us a lot to talk about at home. (…) [We give] cues to each other (…) [As] scenarios with our children happen, (…) we look at each other and (…) give the [flipping lid] hand sign.” (FG1)*
BBT program feedback and contextualization	BBT supports CMF resilience-building	*“[I]n the military, the [SMs] get resilience-training, but not (…) our spouses. [They] have to have a lot of resilience too. Your husband is gone for, however, long.” (FG1)*
	Peer connection during BBT was essential	“*I really connected with some of the people in our group. (…) [I]t was nice to see other parents who are doing and being and struggling with the same things.” (FG6)* *“I would’ve liked if my husband could come.” (…) I feel like I’m trying not to criticize him, like “oh this is what we’re trying to do now”.” (FG4)*
	BBT content was valuable	“*I like the last session (…) focused on the kids versus the adults, but I think having that background of looking at yourself first before you just start putting everything on your parenting skills.” (FG4)*
	BBT requires adaptation to fit CMFs unique challenges	*“[T]he member coming back has to adjust to the child being different, new developmental stages, different perspectives, interest.” (FG2)* *“I use breathing (…) daily. (…) [W]e learned it going through basic [training] (…) [It’s] a continuation.” (FG5)*
	BBT provides flexible program delivery	“*It was broken up and laid out well. (…)[G]o through a couple slides, watch a video, do a couple scenarios. (…) [T]here wasn’t time to get bored. A new way of thinking and teaching; (…) really interactive. I liked that.” (FG2)* *“Honestly, you just need that week for integration.” (FG4)*
MFRCs as community resilience hubs	MFRCs are well-established and trusted by CMFs	*“It’s nice having it at the MFRC. I think everyone is pretty comfortable walking in the doors of the MFRC.” (FG1)* *“[I]t was really great to have members of the MFRC present. [I]f it wasn’t, I think it would’ve been very inaccessible. (…) [T]hey know the lifestyle.” (FG2)*
	BBT may address a CMF resilience service gap	*“[R2MR] was just a 2-h evening (…) - it was still helpful, but [BBT] definitely covered a lot more (…) from the spouse family side.” (FG2)*
	MFRCs partner with CFMWS, PSP, RCChS, and Health Promotions	“*I would love to see so many new people [take BBT]. Could it be run like the PSP programs? (…) health promotions? (…) [SMs] can get the time off work; (…) if they take a health promotion course, they get credits.” (FG4)*

### Theme I: Military Parent Resilience-Building

#### Resilience Is a Learned Skill

BBT reinforced ongoing resilience skill-building. “*We noticed in our group (…) how our thoughts automatically go in one direction instead of focusing on strengths. (…) [W]e definitely have stuff to learn.” (FG1) “There is a lot of improvement I can look forward to (…) Doing it all the time (…) will be really helpful long-term” (FG4).*

#### Resilience Requires Reflection and Self-Awareness

Participants valued taking time to reflect and came to new realizations: *[I]t’s not changing your kid, it’s changing yourselves” (FG4)* and *“I thought I didn’t have any [shoulds/expectations] anymore. (…) [I]t was like a lightbulb just to look at myself. I’ve already seen a shift in the last couple of weeks” (FG1).*

#### Resilience-Building Requires Practice

BBT emphasized the importance of intentional, routine practice. *“[Knowing resilience skills] doesn’t mean I do those things regularly. It’s practicing them, having it re-introduced in different ways” (FG2).* Improvements in self-regulation and mindset shifts were reported: *[Stop] before it escalates. [L]et go (…) and then come back to it” (FG4). The most impactful thing was changing [my] frame of mind” (FG1).* Participants described having to “*[R]e-write the script in my head. (…) [I’ll] really try to be purposeful in how I’m responding” (FG3).* Participants also commented on self-care stating, *“You can learn to care for a baby, but you’ve got to learn to care for yourself” (FG5).*

### Theme II: Military Family Resilience-Building

#### BBT Supports Development of Collective Family Resilience

Participants recognized intergenerational impacts stating: *“[Y]ou learn parenting from your parents. (…) It’s mind-blowing when you realize that you’re raising your child in how to parent” (FG2)*. Collective family resilience and deeper connections in parent and parent-child relationships can be fostered through focusing on resilience and making a shared effort.

#### Resilience Requires Reflection on Parenting Practices

Allocating time to discuss parenting was seen as essential. Participants challenged assumptions about themselves and their children, and validated improvements. *“A significant change in our house is that immediate reaction. [L]ots of breathing, focusing on the positive” (FG4)*; *“I like considering the thinking habits and where we get stuck as parents” (FG1).*

#### Requirement of Collective Understanding and Practice of Resilience Skills

*“[BBT] puts us on the same page” (FG3).* Resilient-thinking helps CMFs respond to challenges, and *“Helps us see things ahead of time” (FG2)*. Participants appreciated the importance of role-modeling, stating

*“[W]hen your kids are little (…) you talk to them all the time. ‘Oh mom is doing this’. [N]ow they’re older, you don’t do that. (…) I actually did it a couple times while [my daughter] was having some meltdowns. [I said], ‘ok, Mom’s going to take a couple deep breaths’ (…) and she just looked at me; (…) she stopped crying” (FG1).*

Emotion and expectation management were also key areas participants addressed.

*“I have to separate myself from work. (…) [While] it might’ve been a hard day at work, it might’ve been a completely happy day at home.” (FG3)*

*“I didn’t need to get angry (…) I can make a choice and just take a breath and be calm and (…) make (…) choices together without it [being] a negative experience” (FG6).*

### Theme III: BBT Program Feedback and Contextualization for CMFs

#### BBT Supports CMF Resilience-Building

While SMs receive resilience-training, CMFs do not. BBT addresses the need and allows SMs and their partners to jointly participate in programs.

#### Peer Connection During BBT Was Essential

Participants valued peer support, stating “*[F]or guys to see other guys having these discussions, and being a part of a parenting group. [T]hat is really valuable.” (FG3)* Those attending individually also indicated that it would be helpful if their partner had access to materials. This would facilitate conversation and enhance consistency in co-parenting. “*[G]ood [for] members and spouses [to have] conversations” (FG3).*

#### BBT Content Was Valuable

Participants appreciated BBT’s focus on enhancing military-promoted skills*: “[W]e learned it going through basic [training] (…) it’s just a continuation.” (FG5)* and sharing knowledge with spouses: *“[T]he take home stuff was nice. [M]y husband can circle his stuff and we talked about it” (FG1).* They also valued the trauma-informed approach, *“[It’s] not ‘I’m a good or bad parent’, it’s ‘what is going on with me?”’ (FG4)*. One participant suggested that BBT-training be offered to SMs post-injury [e.g., Post-Traumatic Stress Disorder (PTSD) or an Operational Stress Injury (OSI)] to help them relearn resilience and help them engage with their children.

*“For someone like me with PTSD, [I] had to be retaught resilience. [BBT] reinforced (…) using resilience. So I (…) had to relearn resilience for myself and then (…) learn it for a child. That was (…) huge. (…) I think the course should be part of [recovery].” (FG6)*

#### BBT Requires Adaptation to Fit CMFs

*“[BBT’s] general coping skills. (…) It’s definitely not tailored [to military], but still helpful” (FG2).* Participants recommended that the BBT program be adapted to address, “*unique challenges to military families” (FG2).* The unique challenges include frequent deployment, relocation, postings, sometimes with limited notice for preparation to occur. CMFs are also frequently isolated from extended family because of being posted across the country, resulting in a subsequent need and emphasize of relying on other CMF community members. Contextualization would also need to highlight helping children adjust to work-related absences, as, *“[Kids] have to re-adjust their thinking (…) - their anger toward that parent being gone” (FG2).* Since the BBT program has been used in civilian groups made up predominantly of mothers, it will be important to ensure better representation with “*More mixed gender videos (…); more dads” (FG2).*

#### BBT Provides Flexible Program Delivery

While participants appreciated BBT’s incorporation of different learning methods, they noted that BBT is information-heavy, and that *“[H]aving online accessibility, spacing out a little bit more, having time for application and integration” (FG4)*, would be beneficial. Additionally, given the struggles for CMFs to have both parents at home (i.e., not away on training or deployment) consideration of synchronous and asynchronous as well as blended delivery formats was suggested to allow for deployed military parents to participate.

### Theme IV: MFRCs as Community Resilience Hubs

#### MFRCs Are Well-Established and Trusted by CMFs

MFRCs provide CMF culture- and circumstance-specific social-emotional supports and services. The MFRC, “*[F]eels like a family setting. So, it was more of a conversation” (FG1). “[I]t’s a military thing. We’re a little more blunt and open to share. (…) [I]t’s ok [at MFRCs] to talk about not being perfect and having struggles” (FG4).* The facilitator’s understanding of military culture was critical to making the content relatable.

*“[O]ne of our examples (…) was ‘it’s not a big deal to be late’. (…) [F]or civilians that’s true, but for someone in the military (…), that is not okay, (…) [MFRC staff] have a unique skill set where they can relate and use the language ‘cause it’s a different language and different culture” (FG1).*

#### BBT May Address a Service Gap Regarding CMF Resilience-Building

While current CAF programs aim to enhance mental health awareness and help-seeking (e.g., R2MR), participants appreciated BBT’s psychoeducational approach, resilience-specific training, and peer-support approach: “*[BBT] definitely covered a lot more than (…) R2MR that I did as a spouse” (FG2).*

#### MFRCs Partner With CFMWS, PSP, RCChS, and Health Promotions

MFRCs’ pre-existing partnerships may facilitate integration of BBT into CAF programming (e.g., InterComm, Sentinel Program, Resilient Relationships).

## Discussion

This pilot study evaluated the suitability of BBT for potential implementation at MFRCs. Specifically, the research team examined whether BBT: (1) facilitates CMF resilience-building, (2) can be contextualized for CMFs, and (3) supports MFRCs in cultivating a culture of resilience. Participants reported that BBT enabled them to focus on personal and family resilience, facilitated an understanding of resilience, fostered open dialogue with peers and partners and enabled intentional role-modeling of resilience.

As the backbone of the CAF, CMFs need to be resilient. Exposure to military-related stressors with adequate resources and skills can allow them to prepare for and thrive in CMF-life (Cramm et al., 2018). Enabling adults to model resilience to children can foster collective well-being, confidence, and resilience. Further, family support and training encourage development of resilience in children 0–8 years during a critical developmental period where families face increased vulnerabilities due to environmental stresses (Cramm et al., 2018). A service gap, however, exists regarding evidence-based programs available through MFRCs that facilitate resilience-building for CMFs.

Centred within military communities MFRCs are well-suited, situated, and trusted to deliver resilience-training to CMFs that both complements CAF initiatives and priorities and is culturally sensitive – a factor known to enhance engagement (Weir et al., 2019). Including programs such as BBT in MFRC programming may not only address the needs of young CMFs but the CAF at large through assisting individual development of resilience skills that can be modelled within families and the extended community. Incorporation of CMF-specific BBT peer groups may also support resilience-building and foster CMF community connection. BBT was also found to align with CAF’s resilience-building initiatives and priorities as outlined in SSE and the Comprehensive Military Family Plan, and may address an MFRC service gap, while complementing program offerings by MFRCs, PSP, RCChS, and Health Promotions.

Canadian military families (CMF)-specific contextualization of BBT and delivery in an online format (i.e., synchronous, asynchronous, and blended) would make the program more accessible to CMFs. Providing alternate scheduling and means of accessing BBT would enhance CMFs program participation. While face-to-face 2-h sessions for 10 weeks is considered optimal, participants indicated that the 3- and 6- session formats were acceptable. Attendance of CAF SMs might increase if BBT were integrated in PSP, RCChS and Health Promotions programming, thereby allowing SMs to attend as a work requirement with the support of their Chain of Command. Further, acceptance of online or blended delivery options has increased. Such formats may enable CMFs in Canada and abroad to equally access programs, thereby enabling MFRCs to have a broader reach.

Cultural alterations would provide the opportunity for participants to reflect on specific changes to family belief systems, organizational patterns, support systems, or communication constraints related to military experiences. While BBT intends to allow for the development of positive outlooks, communication strategies, and understanding of beliefs, development of examples and content reflective specific to military experiences, including frequent relocation and parental absences and risk of work-related illness and injury, would enhance usability with CMFs. Since BBT has been developed and evaluated with civilian populations, such adaptations to better reflect the realities of CMF life will be important to facilitate its widespread usage. The positive response that this pilot of the original BBT program (i.e., without specific military adaption) has received speaks to the promise of the intervention in the context of the MFRCs.

Further identification and evaluation of programs such as BBT is required to ensure that MFRCs deliver a range of resilience-building supports and services in a variety of formats. In partnership with PSP and the RCChS, a CMF-version of BBT could be implemented in English and French through the 32 MFRCs across Canada. BBT would align with MFRCs’ focus on offering evidence-based or standardized programs for which employees can be certified as Master Trainers who could train other employees. Use of an implementation framework would be advisable if nation-wide adoption were considered.

### Future Research

BBT’s effectiveness in general and CMF-specific versions (co-designed with CMFs) require further study, as does evaluation of various models, formats (e.g., virtual, in-person, and blended) and doses of delivery, the long-term impact of resilience-training on parents (solo- and co-parents), children and the family unit, and peer support component. Program delivery by professionals and CMF members, as well as the implementation, spread and sustainability of BBT by MFRCs also warrant further research.

### Limitations

Notable limitations include the limited sample size and representation, with most participants being female spouses of male SMs or Veterans, attending individually. All participants were over 30 years of age, missing the younger demographic. As the delivery schedule of BBT was adjusted to accommodate CMFs, fidelity to the BBT program cannot be claimed and findings cannot be accurately compared to the original BBT. Further, the realities of deployment and the 2020 COVID-19 pandemic added unanticipated complexities.

## Conclusion

This study aimed to examine military parents’ experience of the BBT resilience-training and its impact on CMF resilience. Participants found BBT effective at fostering skills, and promoting a common language, understanding and practice of individual and collective CMF resilience. Program contextualization for CMFs and delivery in an online format would make it more accessible. Scalability across MFRCs and CAF would support efforts to enhance CMF resilience and enrich a culture of resilience. The program aligns with CAF’s resilience-building priorities for SMs and CMFs and may support SMs’ ability to be mission-ready and focused. Building CMF resilience at MFRCs using a program such as BBT offers a template that can be applied to civilian families who also require resilience in everyday life and during stressful or unprecedented times (e.g., family crises, natural disasters, anthropogenic hazards or pandemics).

## Data Availability Statement

The raw data supporting the conclusions of this article will be made available by the authors, without undue reservation.

## Ethics Statement

The studies involving human participants were reviewed and approved by the University of Alberta Research Ethics Board. The patients/participants provided their written informed consent to participate in this study.

## Author Contributions

SB-P, AP, JO, PS, and CM conceived, designed, and conducted the study. All authors drafted, revised, and approved the final manuscript submitted for publication.

## Conflict of Interest

CM was employed by the local MFRC at which the pilot study occurred. The remaining authors declare that the research was conducted in the absence of any commercial or financial relationships that could be construed as a potential conflict of interest.
